# Midwifery-led antenatal care models: mapping a systematic review to an evidence-based quality framework to identify key components and characteristics of care

**DOI:** 10.1186/s12884-016-0944-6

**Published:** 2016-07-19

**Authors:** Andrew Symon, Jan Pringle, Helen Cheyne, Soo Downe, Vanora Hundley, Elaine Lee, Fiona Lynn, Alison McFadden, Jenny McNeill, Mary J Renfrew, Mary Ross-Davie, Edwin van Teijlingen, Heather Whitford, Fiona Alderdice

**Affiliations:** Mother and Infant Research Unit, University of Dundee, Dundee, DD1 4HJ UK; School of Nursing & Health Sciences, University of Dundee, Dundee, DD1 4HJ UK; NMAHP Research Unit, University of Stirling, Stirling, UK; School of Health, Brook Building, University of Central Lancashire, Preston, PR1 2HE UK; Centre for Midwifery, Maternal & Perinatal Health, Faculty of Health & Social Sciences, Bournemouth University, Bournemouth, BU1 3LH UK; School of Nursing and Midwifery, Queens University Belfast, Belfast, BT9 7BL UK; Maternal and Child Health, NHS Education for Scotland, Edinburgh, EH3 9DN UK

**Keywords:** Maternity care, Pregnancy, Care model, Quality, Characteristics of care, Midwifery-led, Randomised controlled trial, Outcomes

## Abstract

**Background:**

Implementing effective antenatal care models is a key global policy goal. However, the mechanisms of action of these multi-faceted models that would allow widespread implementation are seldom examined and poorly understood. In existing care model analyses there is little distinction between *what* is done, *how* it is done, and *who* does it. A new evidence-informed quality maternal and newborn care (QMNC) framework identifies key characteristics of quality care. This offers the opportunity to identify systematically the characteristics of care delivery that may be generalizable across contexts, thereby enhancing implementation. Our objective was to map the characteristics of antenatal care models tested in Randomised Controlled Trials (RCTs) to a new evidence-based framework for quality maternal and newborn care; thus facilitating the identification of characteristics of effective care.

**Methods:**

A systematic review of RCTs of midwifery-led antenatal care models. Mapping and evaluation of these models’ characteristics to the QMNC framework using data extraction and scoring forms derived from the five framework components. Paired team members independently extracted data and conducted quality assessment using the QMNC framework and standard RCT criteria.

**Results:**

From 13,050 citations initially retrieved we identified 17 RCTs of midwifery-led antenatal care models from Australia (7), the UK (4), China (2), and Sweden, Ireland, Mexico and Canada (1 each). QMNC framework scores ranged from 9 to 25 (possible range 0–32), with most models reporting fewer than half the characteristics associated with quality maternity care. Description of care model characteristics was lacking in many studies, but was better reported for the intervention arms. Organisation of care was the best-described component. Underlying values and philosophy of care were poorly reported.

**Conclusions:**

The QMNC framework facilitates assessment of the characteristics of antenatal care models. It is vital to understand all the characteristics of multi-faceted interventions such as care models; not only what is done but why it is done, by whom, and how this differed from the standard care package. By applying the QMNC framework we have established a foundation for future reports of intervention studies so that the characteristics of individual models can be evaluated, and the impact of any differences appraised.

## Background

Provision of effective maternity care is a vital global policy goal as governments seek not only to reduce mortality and morbidity rates [1] but also to ensure that maternal and newborn health and wellbeing are improved. Recent Cochrane reviews of antenatal care models by Dowswell et al. [2] and Sandall et al. [3, 4] have been complemented by an international drive to focus on those characteristics of care which promote the best outcomes [5, 6]. The new ‘lens’ that has resulted provides a mechanism to enable the assessment of maternal and newborn care provision in diverse settings around the world.

Antenatal care varies within and between countries, reflecting different healthcare and political realities. Within many high-income countries the growing acceptance of women’s rights to choice and autonomy in healthcare has led to the growth of a more woman-centred approach to antenatal care. Various ‘alternative’ forms of care have been tried, including ‘continuity of care’ models, where all the professionals involved “share common ways of working and a common philosophy” [7] and ‘continuity of carer’ models in which the same health professionals provide care throughout – which might mean throughout a specific episode, such as labour.

The delivery and scope of antenatal care can affect the health and well-being of women and infants [8, 9]. Different models of antenatal care have been shown to improve maternal and neonatal outcomes, including reduced preterm birth rates and higher breastfeeding initiation rates [10, 11]. Davis-Floyd et al. [12] claim that an over-medicalised approach which promotes the use of routine clinical interventions without a robust evidence-base is counter-productive: the association between unnecessary clinical interventions and increased morbidity is well evidenced [13]. A persistently high caesarean section rate causes concern about various morbidities, including poor neonatal respiratory function and maternal haemorrhage, anaemia, infections, and future placenta praevia/accreta [14]. Downe [15] notes a growing multi-disciplinary evidence base which associates intrapartum interventions with an increased risk of longer-term non-communicable autoimmune disorders, diabetes and even certain cancers [16].

Poor clinical and psychosocial outcomes, for example preterm birth and its associated stresses for the family, represent a considerable burden on personal, familial and society-wide levels [17]. Poor outcomes also lead to significant organisational and financial burdens on health services [18]. Identifying mechanisms which could mitigate or prevent poor outcomes would offer significant benefits to individuals, families, health systems, and societies. Models which positively promote well-being have the potential to produce widespread benefits.

The drive to empower the service user has been a particular focus for maternity care in the UK [19]. The implicit assumption behind this is that informed decisions are negotiated with the health care provider once the woman has had the opportunity to consider the available evidence. High level guidelines (e.g. [20]) collate evidence and grade the evidence level to help this process. Women’s experiences are also now evaluated regularly [21, 22]. There has been significant emphasis in several countries, including the UK, Australia and Canada, on the provision of midwife-led and women-centred care, which can be delivered in a range of ways and situations [3]. Inevitably, these ‘models of care’, which differ from standard care in many countries in which an obstetrician is usually the lead professional, but in which women still receive care by midwives, reflect varying clinical and other situation-specific realities, including midwife-led care primarily for women deemed to be of low-risk, and obstetric-led care for women of all risk levels. While many criticise the conceptual basis, risk considerations permeate the practice and reporting of clinical care and we follow that convention here as it enables consistent reporting of current research. Sandall et al.’s Cochrane review on midwife-led continuity models [3] highlighted the possibility that adverse outcomes, including preterm birth, could be reduced by altering the standard antenatal care package to include improved continuity of care led by midwives. However, questions remain about the causal mechanisms underlying improved outcomes. Research reports may relate *what* is done and *by whom*, but not explain *why* or *how.* Providing additional preventive or supportive care by one caregiver, or a small number of caregivers, may be a factor. McLachlan et al. [23], for example, concede that they could not tell which aspect of their new care model caused the difference in outcome. Further exploration is needed in relation to the full spectrum of models of care to inform both the implementation of effective care models and future research.

The McTempo (Models of Care: The Effects on Maternal and Perinatal Outcomes) collaboration is a multi-disciplinary and multi-institutional research grouping that has been formed to explore and evaluate different care models used in maternity care. As a first step in a planned programme of work to explore what actually makes the difference in these antenatal care models, we conducted a systematic review of randomised controlled trials (RCTs) and systematic reviews (SRs) of RCTs. While we focus on antenatal care in the first instance, we will incorporate intrapartum and postnatal care aspects in future work, as mothers and infants are inevitably affected by the whole continuum. In this paper we report on the midwifery-led models we identified. We have used the term ‘midwifery-led’ rather than ‘midwife-led’ because we anticipated that some of the interventions, while constituting midwifery care, would involve care given by practitioners other than those holding an internationally-recognised qualification as a midwife. We mapped reports of the intervention and control arms of these studies to the evidence-based Quality Maternal and Newborn Care (QMNC) framework developed by Renfrew et al. [5]. While we acknowledge that the control arm in a trial also represents an intervention, in this paper we use ‘intervention’ to refer to the experimental arm of the trial. The QMNC framework offers the opportunity to explore the characteristics of care throughout pregnancy, labour and the postnatal period, and thereby to help to identify the active mechanisms that improve outcomes. In so doing in this initial review we hope to open up the ‘black box’ of antenatal care to identify those constituent parts which make care effective or ineffective.

**Fig. 1:**
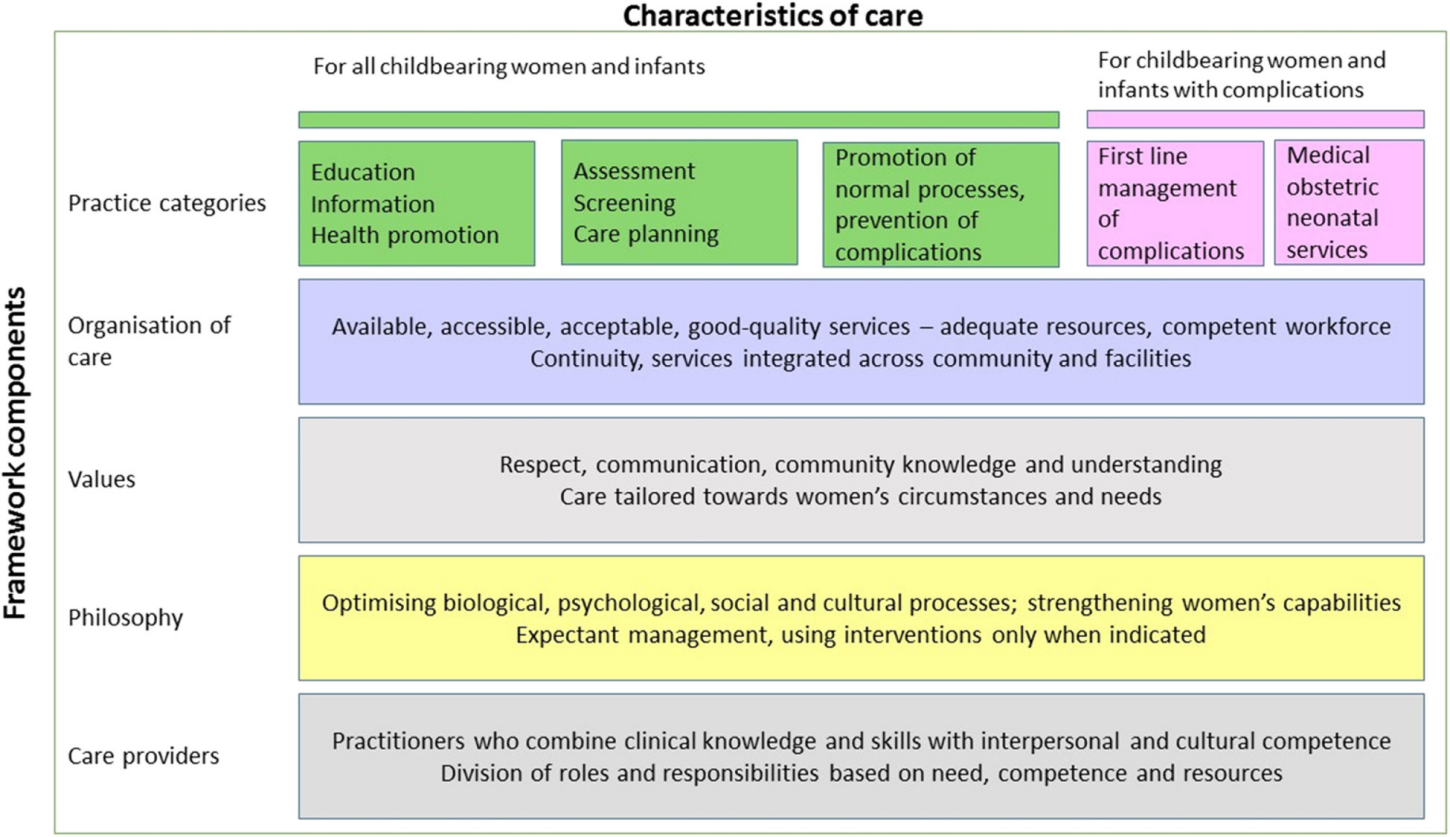
The Lancet Series on Midwifery: Framework for Quality Maternal and Newborn Care (QMNC): Renfrew et al. 2014 [5]

To help us do this we devised a data extraction form based on the QMNC framework [5] (Fig. 1).

The QMNC framework was developed through a combination of conventional systematic review and advances in methods for interpretive synthesis, and included expert opinion input from 35 Lancet Series co-authors and a further dozen or so critical readers. The detailed evidence base used to develop the framework included 461 Cochrane reviews of interventions, 13 meta-syntheses of qualitative studies of women’s views and experiences, and seven reviews of workforce issues. The framework focuses on the needs of women, infants and families across the continuum of care, and can be used to assess the key concepts of quality maternal or newborn care provision by a wide range of providers in diverse care settings worldwide.

The framework identifies five components which are used to organise specific characteristics across the continuum of maternal and newborn care from pregnancy to postpartum and the early weeks of life. These components are: practice categories, organisation of care, values, philosophy, and care providers. The term ‘Practice categories’ comprises five sub-categories, the first three of which are supportive/preventive and are needed by all childbearing women and infants (education, assessment and the promotion of normal processes), with the last two relevant for women and infants with complications (first line management and specific obstetric and neonatal services). Within each of these components, characteristics of care that women and infants need are specified. The framework can therefore be used to identify and then review the scope and content of all maternal and newborn care. In this paper we use it for one specific purpose - to analyse trials of midwifery-led care.

While the scope of midwifery practice varies worldwide, its core characteristics can be identified within the framework, which thus provides a means of evaluating reports of midwifery care interventions internationally. The McTempo project will in future identify not just the continuity models included in the recent Cochrane review, but a wide range of specific antenatal care models which have been the subject of RCT evaluation. For the work reported here we limited ourselves to examining in detail those studies where the intervention was deemed to be ‘midwifery-led’. We are also limiting ourselves to original reports of RCTs in order to assess systematically the reporting in the current literature of the characteristics identified by the QMNC framework. The aim of this paper therefore is to map the characteristics of antenatal care models tested in Randomised Controlled Trials (RCTs) to a new evidence-based framework for effective maternal and newborn care.

## Methods

Inclusion/exclusion criteria were developed using a PICOS format (Population, Interventions, Comparator, Outcomes, Study design). Primary searches were conducted by the York Health Economics Consortium during July–August 2014. The main structure of the search strategy comprised three concepts: Antenatal care; Models linked to carers – non-midwife specific; Models linked to midwives. These concepts were combined as follows: (Antenatal care AND Models linked to carer – non-midwife specific) OR Models linked to midwives. In order to retrieve studies missed by this approach, the strategy also included additional focused stand-alone lines on terms that were potentially relevant for antenatal care. The search was restricted to primary descriptions of RCTs (primary and sibling papers) published in the English language in order to identify the salient features of the interventions. SRs were not included within the context of this part of the research because they are one step removed from the context of the interventions themselves. No time limits were used.

A preliminary screening of titles and abstracts by the McTempo team members identified those papers required for full assessment. This involved independent scrutiny by paired members of the team using the SPIO formula:

- Study design - RCTs
- Population - Women receiving antenatal care (+/− family); health care professionals/lay people planning/delivering such care
- Intervention - Models of antenatal care (midwifery/group/obstetrician/family doctor/shared/peer/birth attendant/doula)
- Outcome(s) - Maternal/infant perinatal outcomes; maternal psychosocial outcomes; organisational outcomes, including economic evaluations; maternal health behaviour outcomes.

We operationalised the analysis using the QMNC framework by listing the characteristics of care incorporated in the five framework components (practice categories, organisation of care, values, philosophy, and care providers). We then applied this to the 17 studies that we identified (see Results section). We assessed the presence or absence in the main article reporting the RCT and in the identified ‘sibling papers’ of these 16 stated characteristics (listed in Table 1) using a scoring system: 0 = no or very minimal reference; 1 = some reference, but not fully explicit; 2 = explicit reference. Across the 16 characteristics this gave a maximum possible score of 32. The intervention and control arms were assessed separately. Assessments were made independently by paired members of the McTempo team, and then cross-checked for discrepancies. If necessary, a third member of the research team reviewed the scoring and made a final decision.

**Table 1:** Overview of 17 RCTs of midwifery-led care

				Care provision		
Study: First author; main article publication date (+ any subsidiary papers); Data collection years	Country; number of intervention participants (n=); (+ sites involved in study)	Study participants: n=; characteristics	Brief intervention details	Antenatal	Intra-partum (inc. immediate post-partum day	Post-partum, beyond day of birth
1.Begley et al. 2011 [67] Data collection 2005–07	Ireland; two midwifery units (Drogheda, Cavan)	n = 1102; Healthy pregnant women (i.e. low risk)	Midwifery-led care by same small team of midwives (7 midwives in one unit/team, 12 in the other unit) for the antenatal period, intra-partum and up to 7 days post-partum	✓	✓	✓(7 days)
2.Biro et al. 2003 [26] (+Biro 2000) [41] Data collection 1996–98	Australia; one medical centre (Melbourne)	n = 502; Pregnant women of any risk status	Team midwifery provided by 7 midwives for antenatal, intra-partum and the immediate post-partum period (1 day)	✓	✓	✓(1 day)
3.Flint et al. 1989 [27] Data collection 1983–85	UK (England); one maternity hospital (London)	n = 503; Low risk pregnant women	Team of 4 midwives offering continuity of care for antenatal, labour and immediate post-partum period (exact period not specified)	✓	✓	✓(unspecified)
4.Giles et al. 1992 [28] Data collection 1989–90	Australia; one teaching hospital (Sydney)	n = 43; Low risk pregnant women	Midwife-led care from team of 4 midwives throughout pregnancy (labour and post-partum care was provided by other staff/midwives)	✓	???	
5.Gu et al. 2013 [29] Data collection 2011	China; one obstetric hospital (Fudan)	n = 55; Low risk, first pregnancy/birth	Midwife-led antenatal, intra-partum care, and for first two hours post-partum provided by one of 10 midwives (or an associate)	✓	✓	
6.Harvey et al. 2002 [30] Data collection period not stated	Canada; one tertiary referral centre (Alberta)	n = 101; Low risk women/pregnancies	Midwife-led care by team of 7 midwives, from booking visit through to intra-partum and post-partum, plus a 6 week follow-up clinic visit	✓	✓	✓(one 6 week follow-up visit)
7.Hicks 2003 [31] Data collection period not stated	UK (England); antenatal clinics in study area (location not stated)	n = 200; First 200 low risk women to book in study area once study began	Team midwifery (eight midwives) providing continuity of care	✓	✓	✓
8. Homer et al. 2001a [68] [BJOG] (+Homer et al. 2001b [37] [AHR]) Data collection 1997–98	Australia; one teaching hospital (Sydney)	n = 550; Women with no significant medical problems or previous caesarean (i.e. low risk)	Community-based continuity of midwifery care through a team of 6 midwives and one obstetrician; intra-partum care and 3–4 domiciliary visits in post-natal period	✓	✓	✓(3–4 visits)
9.McLachlan BK et al. 2000 [40] Data collection period not stated	UK (England); 35 GP practices across six areas (North Staffordshire)	n = 770; Any pregnant women in study area	Caseload midwifery – midwives working in groups of 2–3 to achieve high degree of continuity with community focussed care for pregnancy and delivery in hospital. No community follow-up specified	✓	✓	
10.McLachlan HL et al. 2012 [23] Data collection 2007–11	Australia; one tertiary hospital (Melbourne)	n = 1156; Low risk women/pregnancies	Caseload midwifery – one primary midwife with back-up midwives. From booking visit until birth, and early post-natal hospital stay (approx. 1–3 days).	✓	✓	✓(1–3 days in hospital)
11.Rowley et al. 1995 [48] Data collection 1991–92	Australia; one tertiary university hospital (NSW)	n = 405; High or low risk women/pregnancies	Team midwifery from 6 midwives for antenatal period until delivery and ‘just after’ birth	✓	✓	
12.Tracy et al. 2013 [32] Data collection 2008–11	Australia; two teaching hospitals (NSW and Brisbane)	n = 871; Pregnant women with any risk: singleton pregnancy and no planned caesarean (other risks acceptable)	Caseload midwifery from named midwife or back-up midwife, giving antenatal, intra-partum and post-natal care (up to 6 weeks after birth)	✓	✓	✓(up to 6wks)
13.Turnbull et al. 1996 [65] (+Young 1997 [69], Shields 1998 [70], Turnbull 1999 [71]) Data collection 1993–94	UK (Scotland); One maternity hospital (Glasgow)	n = 648; Low risk women/pregnancies	Midwife-led care with continuity of carer (named midwife with back-up midwife), throughout antenatal, intra-partum and post-natal period (women seen at home, but length of follow-up not specified)	✓	✓	✓(unspecified)
14.Waldenström et al. 2000 [72] (+Waldenström 2001 [73]) Data collection 1996–97	Australia; one women’s hospital (Melbourne)	n = 495; Low risk women/pregnancies	Team midwifery (8 midwives) providing continuity of care from booking visit, to birth, and post-natal ward (days 1–3, in hospital)	✓	✓	✓(1–3 days in hospital)
15. Waldenström et al. 1994 [35] (+Waldenström 1997a Birth [74], Waldenström 1997b BJOG [38]) Data collection 1989–93	Sweden; one birthing centre (Stockholm)	n = 928; Low risk women/pregnancies	Team midwifery (10 midwives) providing antenatal, intra-partum, and post-partum care (up to 2 months after birth)	✓	✓	✓(up to 2 months)
16.Walker et al. 2013 [33] Data collection 2009–10	Mexico; 27 rural clinics (Oaxaca and Guerrero states)	n = 461; All pregnant women in study area	Team of 12: obstetric nurses (4) and midwives (8) added to rural practice care for antenatal, intra-partum and post-natal period (length of follow-up not specified)	✓	✓	✓(unspecified)
17.Wu et al. 2010 [34] Data collection 2000–3	China; rural community-based model (Anhui province)	n = 673; All pregnant women in intervention areas	Systematic midwifery care during antenatal care and delivery	✓	✓	

## Results

**Fig. 2:**
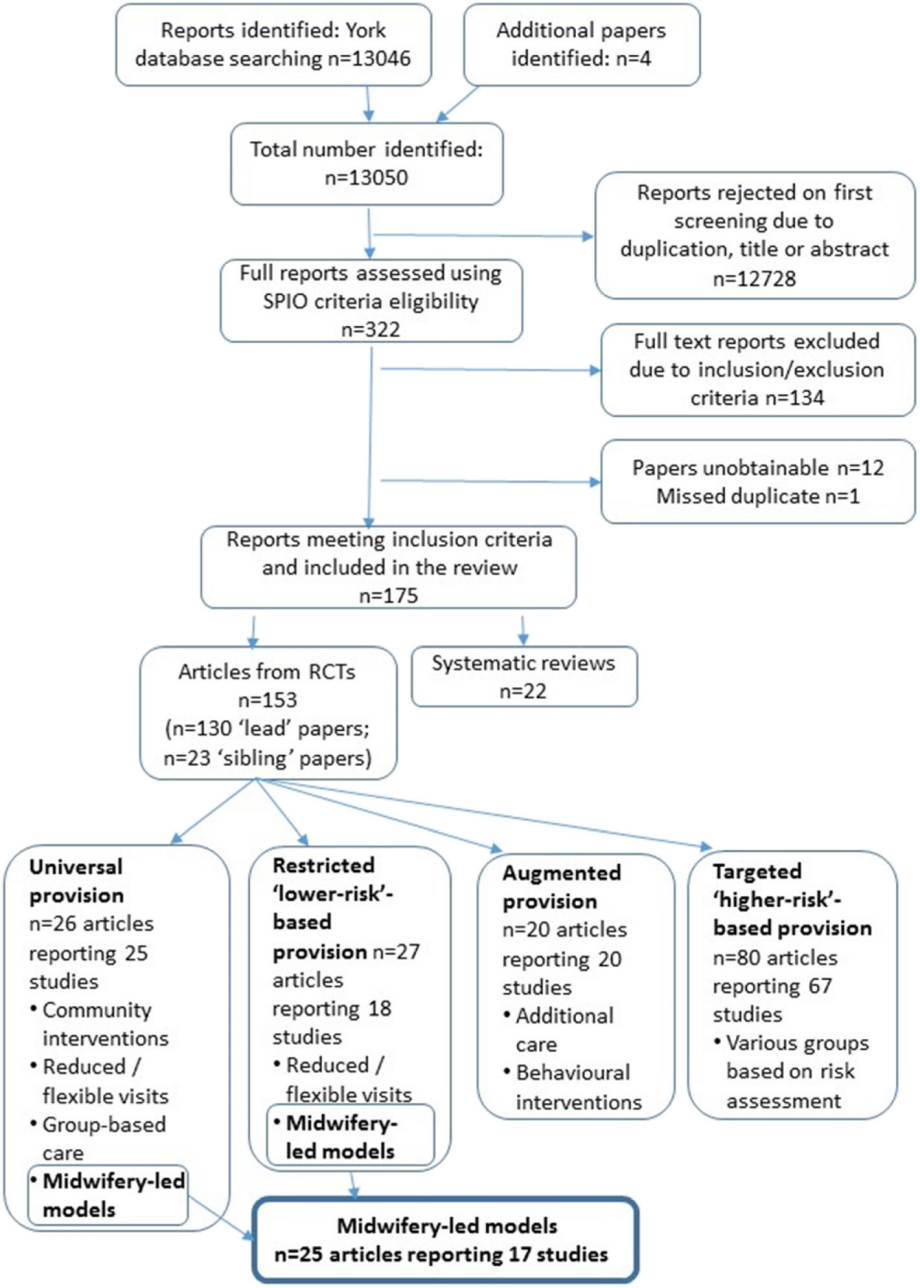
Search and screening process

The initial search identified 13,046 titles, and a further four were identified through the reference lists of included studies. Following de-duplication and screening of titles and abstracts by paired members of the McTempo team, 22 systematic reviews and 153 articles met the inclusion criteria; several individual studies were reported in more than one article. In a separate paper we will report on the categorisation of the studies reported in these 175 citations, but briefly, our taxonomy identified four distinct models: ‘Universal provision’ (for all women irrespective of health state or complications); Restricted ‘lower-risk’-based provision (midwifery-led or reduced/flexible visit approach for healthy women); Augmented provision (antenatal care as in Universal provision above but augmented by clinical, educational or behavioural intervention); Targeted ‘higher-risk’-based provision (for woman with defined clinical or socio-demographic risk factors). From the ‘Universal provision’ and ‘Restricted ‘lower-risk’-based’ provision models we identified 17 midwifery-led RCTs (reported in 25 papers) which are the focus of analysis of this paper (see Fig. 2 for summary of screening and identification of included studies). In these interventions the midwife was the primary caregiver and lead professional with responsibility for the care; obstetric or other medical back-up was available should complications arise. These 17 studies included various forms of care delivery including team midwifery, defined as a group of midwives providing care and taking shared responsibility for a group of women from the antenatal period, through labour and postnatal care [24], and caseload midwifery, characterised by a midwife undertaking responsibility for the continuum of care throughout pregnancy, birth and the postnatal period for a small identified number of women [25].

The review and screening process identified 25 papers within the ‘Universal’ and ‘Restricted ‘lower-risk’-based’ categories that between them reported 17 RCTs involving midwifery-led antenatal interventions. The 17 original studies are detailed in Table 1. This shows the principal report of the RCT and the ‘sibling papers’ identified by our search.

The first study reported was published in 1983, indicating that midwifery-led care has been the subject of research for over 30 years. As indicated in Table 1, most of the studies took place in Australia (*n* = 7) or the UK (*n* = 4); there were also two Chinese studies, and single studies in Ireland, Canada, Sweden and Mexico. Most of the studies (11/17) were restricted to women categorised as ‘low risk’, although the definition of low risk varied between studies. Those not focussed exclusively on ‘low risk’ women were from Australia [26–28], the UK [29], China [30] and Mexico [31]. Our assessment of the presence or absence of reporting of the components and characteristics from the QMNC framework [5] is shown in Table 2.

**Table 2:** Midwifery-led RCTs: level of published evidence in Intervention arm [Control arm] of characteristics of maternity/newborn care identified in QMNC framework

	Key to scores: 0 = not present; 1 = mentioned, not detailed; 2 = discussed in some detail																
	Practice categories			Practice categories		Organisation of care				Values		Philosophy			Care providers		
Author, Year	Education info HP	Assessment screening planning	Normal process, complication prevention	Complication referrals	Medical obstetric neonatal services	Organisation of care	Resources	Competent Sustainable workforce	Integration	Respect, Communicate	Tailoring	Optimising	Women’s capability	Intervention use	Knowledge skills	Roles skill mix	Total scores
1 Begley 2011 [67]	0 [0]	2 [1]	1 [0]	1 [1]	1 [1]	2 [1]	1 [0]	1 [0]	2 [1]	0 [0]	0 [0]	0 [0]	0 [0]	1 [0]	1 [0]	1 [2]	14 [7]
2 Biro 2003 [41]	1 [0]	2 [0]	1 [0]	1 [0]	2 [0]	2 [2]	1 [0]	1 [0]	1 [1]	1 [1]	1 [0]	1 [0]	1 [0]	1 [0]	1 [0]	1 [1]	19 [5]
3 Flint 1989 [27]	1 [0]	0 [0]	1 [0]	1 [0]	2 [0]	2 [0]	1 [0]	2 [0]	1 [0]	1 [0]	1 [0]	1 [0]	2 [0]	1 [0]	0 [0]	1 [0]	18 [0]
4 Giles 1992 [28]	2 [0]	0 [0]	0 [0]	1 [1]	1 [1]	1 [1]	1 [0]	1 [0]	1 [1]	0 [0]	0 [0]	0 [0]	0 [0]	1 [0]	1 [1]	1 [1]	11 [5]
5 Gu 2013 [29]	2 [0]	2 [0]	2 [0]	0 [0]	0 [0]	2 [2]	1 [0]	2 [0]	1 [0]	2 [0]	2 [0]	2 [0]	2 [0]	2 [0]	2 [0]	1 [1]	25 [3]
6 Harvey 2002 [30]	0 [0]	1 [0]	1 [0]	1 [0]	1 [0]	2 [1]	1 [0]	1 [0]	1 [0]	0 [1]	0 [0]	1 [0]	0 [0]	2 [0]	1 [0]	1 [0]	14 [2]
7 Hicks 2003 [31]	0 [0]	1 [1]	1 [0]	1 [0]	1 [0]	2 [2]	1 [0]	1 [0]	2 [1]	2 [0]	1 [0]	1 [0]	0 [0]	0 [0]	1 [0]	0 [1]	15 [5]
8 Homer 2001 [37]	2 [0]	0 [0]	1 [0]	2 [1]	2 [0]	2 [1]	1 [1]	1 [0]	1 [1]	1 [0]	1 [0]	0 [0]	0 [0]	1 [0]	0 [0]	1 [1]	16 [5]
9 McLachlan BK 2000 [40]	0 [0]	0 [0]	1 [0]	2 [0]	2 [0]	2 [2]	1 [0]	1 [0]	2 [0]	0 [0]	1 [0]	1 [0]	1 [0]	2 [0]	1 [0]	1 [1]	18 [3]
10 McLachlan HL 2012 [23]	1 [0]	0 [0]	1 [0]	0 [2]	0 [1]	2 [1]	0 [0]	2 [0]	2 [1]	0 [0]	1 [0]	1 [0]	0 [0]	1 [0]	2 [0]	2 [0]	15 [5]
11 Rowley 1995 [48]	1 [0]	0 [0]	0 [0]	0 [0]	1 [0]	1 [0]	1 [0]	1 [0]	0 [0]	1 [0]	1 [0]	0 [0]	0 [0]	1 [0]	1 [0]	1 [0]	10 [0]
12 Tracy 2013 [32]	2 [1]	2 [1]	2 [0]	2 [1]	2 [1]	2 [2]	1 [1]	1 [1]	2 [1]	2 [1]	2 [1]	2 [1]	1 [1]	2 [1]	0 [0]	2 [1]	27 [15]
13 Turnbull 1996 [65]	1 [0]	2 [0]	1 [0]	1 [1]	1 [1]	1 [1]	1 [1]	1 [0]	1 [1]	2 [0]	2 [0]	2 [0]	2 [0]	2 [0]	0 [0]	1 [0]	21 [5]
14 Waldenström 2000 [72]	1 [0]	1 [1]	0 [1]	1 [1]	1 [1]	1 [2]	1 [0]	1 [1]	1 [0]	2 [1]	2 [1]	0 [0]	1 [0]	1 [0]	1 [0]	1 [1]	15 [10]
15 Waldenström 1994 [35]	0 [0]	1 [1]	2 [1]	1 [1]	1 [1]	2 [2]	2 [2]	0 [0]	2 [1]	2 [0]	1 [1]	2 [0]	1 [0]	2 [1]	1 [1]	1 [1]	21 [13]
16 Walker 2013 [33]	0 [0]	1 [0]	1 [0]	0 [0]	0 [0]	1 [1]	0 [0]	1 [0]	1 [0]	0 [0]	0 [0]	1 [0]	0 [0]	1 [0]	1 [0]	1 [0]	9 [1]
17 Wu 2010 [34]	1 [0]	1 [0]	1 [0]	1 [0]	1 [0]	1 [1]	1 [0]	1 [0]	1 [1]	0 [0]	0 [0]	0 [0]	0 [0]	0 [0]	0 [0]	1 [0]	10 [2]
Studies reporting this characteristic	11 [1]	10 [5]	14 [2]	13 [8]	14 [7]	17 [15]	15 [4]	10 [2]	16 [10]	10 [4]	12 [3]	11 [1]	8 [1]	15 [2]	12 [2]	15 [10]	

Quality scores for the models ranged from 9 to 25 for the midwifery-led interventions (out of a possible 32), with the majority of models reporting fewer than half the characteristics associated with quality maternity care (Table 1). Overall our scoring system found little temporal association with scores, although the two highest scoring studies were published recently [29, 32]. Two other recent studies with low scores were from China and Mexico [33, 34].

### Practice categories

Discussion of what was involved in the studies focussed predominantly on the intervention arm. Even so, only 10 of the 17 studies mentioned the need for the intervention to provide assessment and screening for pregnant women. Most referred to the giving of information and health education, but three did not refer at all to the promotion of normal processes within the intervention arm, and in only three cases was this discussed in any real detail [29, 32, 35]. Similarly, there were gaps in the provision of information about women and infants who developed complications, and the provision of obstetric and neonatal services: Gu et al. [29] and Walker et al. [36] did not refer to these at all. The content of care in the control arms was rarely discussed in any detail, particularly in relation to the provision of care for mothers and infants who did not develop complications.

Most of the reported studies were targeted at women assessed as ‘low risk’, but most still referred to how clinical complications in women within the intervention arm would be managed within the trial. For example, Homer et al. [37] detailed how the community-based team continued to care for women who developed antenatal complications, making transfer to standard care unnecessary. We discuss the implications of the use of the word ‘risk’ in the discussion section.

### Organisation of care

This was the component that was best described. Reports included a discussion of the model itself, and very often its integration with other services, including those in the community. Some studies [32, 38] provided a comparative table detailing the differences between the intervention and control arms, although even these tended to focus on *what* was done rather than *how* or *why*. However, even within the ‘Organisation of care’ component some of the characteristics were much less well described: several studies did not refer at all to the need to have a competent sustainable workforce, for example.

A team midwifery approach was examined in 12 studies (numbers 1–8, 11, 14–16 in Table 1). In the studies included here, care was delivered by a team of between four and 12 midwives in order to provide continuity. Flint et al. [39] concluded that a team of just four would have to be increased to five or six in order to cover the 24-hour period adequately. In the Giles et al. study [28] a team of four midwives provided antenatal care but not labour/birth care.

In four studies (numbers 9, 10, 12 and 13 in Table 1) a caseload approach was adopted. One or more midwives was available for support when the caseload midwife was unavailable due to holidays, sickness, or caring for another woman in labour. This approach aimed to provide continuity of carer as well as care (the distinction is noted in the Background section), on a more one-to-one basis [24]. The women were likely to have met both the caseload midwife and the back-up midwife/midwives during antenatal care, and were therefore unlikely to be attended during labour by an unfamiliar person. Caseload size per midwife ranged from 35–45 women at any one time, with midwives also providing back-up for colleagues [23, 32, 40]. These figures are consistent with, or slightly higher than, those stated by Hartz et al. [25].

The community-based cluster RCT in Wu et al.’s Chinese study [34] resembled a team approach, but details of how this was organised were lacking.

In most cases the continuum of care provided within the identified models included antenatal, intrapartum and postnatal care (Table 1), the latter ranging from one day to up to two months.

### Values and philosophy

The QMNC framework Values component includes respect, communication and community knowledge and tailoring care to individual circumstances and needs. The Philosophy component describes an approach to care which optimises biological, psychological, social and cultural processes, and which avoids unnecessary interventions. This approach also involves recognising and strengthening women’s own capabilities, one aspect of which is respecting women’s rights to engage in decisions about their own care.

Discussion of values and philosophy was missing in almost all of the reports of the control arms, and was very limited in the reports of the intervention arms. Only eight studies referred to the need to strengthen women’s own capabilities, and only ten mentioned the importance of respect and communication. While there was almost no discussion at all in the Flint et al. [39] paper about what the control group received, they did note that an aspect of the intervention was to encourage discussion of anxieties. The intention was to empower women with a sense of being in control, so preparing them more effectively for labour. Biró et al. [26] referred to the team philosophy regarding ‘natural childbirth’ as a possible reason for less intervention and shorter hospital stay. In another paper reporting the same study [41] Biró et al. referred to women saying the team midwives provided better emotional support, and reported the feeling that they were better informed and more involved in decision making.

### Care providers

Twelve of the studies referred to the need for those delivering care in the intervention arm to have adequate knowledge and skills; however only two referred to this with regard to the control arm. The management of skill mix was more adequately covered, at least with regard to the intervention arms. McLachlan et al. [23] claimed that the study site had strong management and organisational support.

## Discussion

The purpose of this study was to help to identify the characteristics of those care models which may result in improved outcomes for mothers and infants. To do this we devised a novel approach to assessing studies using a recently developed evidence-based framework [5]. We acknowledge that our 0-1-2 scoring system is a rather blunt instrument. We mitigated subjectivity by having paired members of the team review each paper independently. An additional challenge was that we applied a recently developed conceptualisation of high quality maternity care to studies that date back some thirty years. Certain features of maternity care may not always have been recognised. Furthermore, an increased awareness over the years of complex interventions and of process evaluation have sometimes been reflected in more detailed reporting of recent trials. However, we feel it is essential to examine the evidence that does exist, and to establish a foundation for the measurement and reporting of specific aspects of care models in future studies. Our intention is to identify what it is that leads to an outcome; this means understanding not just what is done and by whom, but what the underpinning rationale is for the intervention and control arms and how they are made to work in practice. Thus the QMNC framework can be used to assess antenatal care provision, in particular whether a model of care has the characteristics that are likely to result in improved outcomes for mothers and infants. However, determining whether those outcomes are actually achieved in any given setting or model of care, and to which care characteristics they are attributable, will necessitate further study.

The data extraction tool based on the QMNC framework was developed in order to identify those characteristics of maternal and newborn care which feature in reports of RCTs. All but one of the 17 RCTs (Wu et al. being the exception) claimed some clinical, psychosocial or organisational benefit from the intervention, but as we did not perform a meta-analysis of these heterogeneous outcomes we cannot comment on the studies’ actual effectiveness. Nevertheless, this is the first study to attempt systematically to identify and describe the characteristics of care models that may be leading to improved outcomes. Although our literature search identified five associated systematic reviews we did not analyse these here as such reports are one step removed from analysing the component parts of an intervention.

An earlier review of women’s views of community-based maternity care in the UK found that, despite an extensive literature, there are important gaps in the evidence, including information about what is done, how this is done and by whom [2], limiting replication and the generalizability of findings. Although more evidence is now available many of these gaps remain, and the QMNC framework provides a ‘lens’ through which research in maternity care can be evaluated. Describing midwifery interventions and findings in terms of their underpinning values and philosophy, as indicated in the QMNC framework [5], can help to situate and define the factors that may be making the difference. This may be by unpacking the essential contribution that midwives can make and are making to the skilled care of women during pregnancy, childbirth and the postnatal period, and to improving outcomes for mothers and infants alike. Unpacking these attributes is an important step in improving the quality of maternity care for women globally. An example of this is the use of psychosocial support interventions to improve maternal mental health outcomes, as identified in systematic review evidence by Dennis and Dowswell [42], which could be potentially delivered by midwives given their intensive contact with women during pregnancy. However, Alderdice et al. [43] emphasise that further evidence is needed on how midwives contribute to the effectiveness of these interventions.

While reports of RCTs focus more on the characteristics and impact of the relevant experimental intervention, it is just as important to report on the control arm of a study. Given the variety of care models worldwide, and changes over time, it cannot be assumed that readers will necessarily understand the rationale for or workings of what is termed ‘standard’ care. It is important, especially in the main paper from a study, to explain the essential aspects of control and intervention arms so that the fundamental differences between the intervention and comparator can be assessed. While discussion of the control arm was partial or absent in many cases, we note that Waldenström et al. [35] and Tracy et al. [32] did include most of the essential information about this in their papers. In many studies the control arm also included midwifery care, albeit within a model where major decisions about care were taken by doctors. Since midwives provided aspects of care in both arms of many of these studies it is important to be clear about what it is that makes the difference. Details about who and what are insufficient; why and how are also required.

The characteristics were detailed to varying degrees in these studies, with only Biró et al. [41] giving explicit details about all the framework components. Detailing the characteristics of care can help to identify either ineffective practices, or those that interfere unnecessarily with normal processes [5].

Our inclusion criteria meant that our search identified the continuity models included in Sandall et al.’s Cochrane review [3], but it also included other antenatal care models. Most were from Anglophone and/or European countries. A limitation of our study in this respect was the exclusion of non-English language publications. The exceptions (two Chinese studies and one from Mexico) occurred in settings where there is not the same tradition of midwifery as in the countries in which the other studies took place, and it is difficult to make comparisons. Nevertheless, the QMNC framework is underpinned by a global evidence base and the future work of the McTempo collaboration will go beyond midwife-led approaches to examine global antenatal care models.

Funding of antenatal care varies across the world in both high-income and low-income countries; schemes may be funded publicly or privately, or by insurance, or by a combination of these. Most studies did not specify care funding details, although Wu et al. (2013) noted that the collapse of the collective-based medical care schemes in China and the introduction of market reforms in the 1980s meant that farmers now had to pay for health care out of their own pocket. In the Gu et al. (2011) trial, the antenatal intervention model was free of charge during the study period, although women had to pay for perinatal services (personal communication).

### Practice categories

While the characteristics of this component were included in relation to most of the intervention arms, information about the control arms was very limited except for discussion of dealing with clinical complications. However, even within the intervention arms it was surprising to find that several studies presented no discussion of health education or health promotion, or of assessment and screening during pregnancy. It was perhaps particularly surprising that there was little detailed discussion of the promotion of normal processes and the prevention of complications. Promoting normal physiological processes might be thought to be a mechanism that underpins these interventions, and yet with few exceptions there was little discussion of what the models did, or intended to do in this regard. Discussion was most informative in relation to the presence of medical, obstetric or neonatal services – the area which, Renfrew et al. [5] argue, has received disproportionate attention within global maternity care at the expense of the preventive and supportive care for women that minimises complications occurring.

### Organisation of care

We used the QMNC framework to assist with the evaluation of the antenatal care models by providing a structure against which to evaluate the interventions. With regard to the quality of reporting study characteristics, there did not appear to be an association between the year that the studies were carried out, or the country/location, or whether a team or caseload approach was used. Team midwifery, which involves a team of midwives providing care and taking shared responsibility for a group of women for antenatal, intrapartum and the postnatal period, was the predominant model offered in the included studies. The recent Better Births report [44] recommends a continuity of carer model based on small teams of 4–6 midwives, although some debate still exists about the merits and demerits of team midwifery [4]. There may also be a lack of clarity about how responsibility should be shared between the midwives within the teams [45].

With regard to the more one-to-one approach provided by caseload midwifery, the latest NICE intrapartum guidance [20] concludes that the evidence shows that women prefer this model of care to traditional ‘shared care’ whereby care is shared between GP surgery or health centre and the maternity unit [7]. Women receiving caseload midwifery were less likely to have interventions during labour, and there was no evidence of difference or detriment in maternal or neonatal outcomes. However, the distinction between team midwifery approaches and caseload approaches is not always well defined [25]. Although our review was able to distinguish which type of model was being provided in most cases, it is necessary for approaches to be sufficiently described in published papers, to inform assessments and comparisons of outcomes.

With regard to caseload size, there was some consistency within the identified studies, ranging from 35 to 40 women per midwife at any one time. However, these figures do not include requirements to provide back-up for other midwives when needed. Our study was restricted to antenatal models; it is possible that the same factors apply during labour and after the birth. Where caseload figures are higher, there may be cause for concern about whether women in childbirth can be provided with the recommended level of care and support for the full period of their labour [46, 47].

In many of the studies we examined both study arms involved care by midwives. Midwifery care was not therefore being compared with another completely different form of care. This means that any difference between the trial arms reported in the studies is likely to underestimate the impact of care by midwives, since both trial arms received versions of such care. The fact that to date most studies have focused on women assessed as being at ‘low risk’ (variously described, there being no universal definition of risk categories) raises the question of whether midwifery-led antenatal care models can be extended to other women. Two studies in this review suggest that this may be possible without detriment to safety [32, 41]. A further study involving ‘low risk’ and ‘mixed risk’ women reported fewer adverse maternal or infant outcomes [48]. Unnecessary interference with normal physiological processes of childbirth has been shown to increase the likelihood of complications [49]. Our taxonomy (referred to here but reported in detail in an accompanying paper) includes the term ‘risk’, but we are simply reporting the basis on which many antenatal care interventions have been based in the past. This much is inescapable, but we also make the point that we do not wish this terminology to reify the risk-based approach in future interventions or models.

### Values and philosophy

Incorporating values of respectful and individualised care and a philosophy of optimising normal processes and strengthening women’s capabilities into care are essential if high quality care is to be delivered [6]. As noted in our findings, measurement of the perceptions of women and the acceptability of the model/scheme under offer was very limited. The importance of providing care that is tailored to individual needs is now well recognised. In 2014 the World Health Organisation [50] stated that “every woman has the right to the highest attainable standard of health which includes the right to dignified, respectful health care”. It has been found that abusive and disrespectful care is harmful but unfortunately widespread [51, 52]. Freedman and Kruk [53] argue that disrespectful and abusive behaviour “is not the phenomenon of a few bad apples. Rather, it runs wide and deep within the maternity services of many countries.” Respectful and tailored care is important not only because it is desirable and is a human right, but also because it results in improved outcomes: the benefit of promoting childbirth with fewer interventions, including caesarean section, may be of immediate and longer term benefit to both mothers and infants [54]. Indeed, Warren et al. [55] note that reports in Kenya have linked pregnant women’s fears of encountering disrespect and abuse with their non-attendance at clinical facilities. Sacks and Kinney [56] argue that the scene is set for global strategies to be agreed to pursue this agenda. We note that this increased awareness of the need for care to be respectful and aimed at encouraging normal processes should encourage more explicit thinking about the rationale for interventions. In other words, care must be respectful and must also promote normal processes.

### Care providers

With regard to the knowledge and skills of care providers, these can have a significant impact on reducing maternal and infant mortality [5]. Prompt recognition and management of complications are skills that can save lives and lessen subsequent morbidity [57]. This applies across the continuum of care, from pregnancy through to the postnatal period, and the early weeks of life, as use of the QMNC framework in analysing existing studies has shown. However, midwifery is more than a set of basic core skills and competencies [58]; care within the scope of midwives’ practice – as defined by the International Confederation of Midwives [59] – in conjunction with medical and public health colleagues, is associated with better quality care and with sustained reductions in maternal and neonatal morbidity [5]. Having strong management and organisational support, as noted by McLachlan et al. [23], is a vital factor for any organisation, but particularly one which is introducing innovative approaches.

While the main focus of this review is antenatal care, it is evident from the description of interventions detailed here that postnatal follow-up can vary considerably. Although maternity care is often concluded after the 6–8 week postnatal visit in the UK [60], and input may often be concluded earlier than this, many of the studies in our review did not follow women and infants up for this length of time, indicating that continuity of care may not be on offer following birth. Although standardised postnatal midwifery visits were no longer required after 1986 in the UK [60] and are not part of routine care in many other countries, having follow-up from a midwife who has knowledge of the pregnancy and birthing history may help women who have recently given birth to feel more supported during the early days and weeks with their new baby [61]. It may also improve rates of breastfeeding [62, 63] and support the identification and prevention of postnatal depression [64]. Interventions that offer continuity of support beyond birth may therefore improve longer term outcomes.

This review has focussed on care models as they affect maternal and newborn outcomes. Apart from the Turnbull et al. trial [65] none of the included studies investigated the views and experiences of midwives or other staff. Hundley et al.’s study [66] showed a small but significant increase in midwife satisfaction in a midwife-managed delivery unit (the best predictors being autonomy and continuity of carer), but this was not eligible for our review because of the lack of antenatal care involvement. Our planned McTempo research programme will include other outcomes, including staff wellbeing, burnout and organisational and resource-related outcomes.

## Conclusion

Although antenatal care models vary worldwide, the evidence-based QMNC framework allows for the characteristics of different care models to be assessed and compared. Our use of this framework found that although 17 RCTs of antenatal care models have been conducted, there are important gaps in knowledge about the characteristics of those models that have an impact on outcomes. This hampers the ability to replicate these studies or to generalise from their findings and limits implementation.

Any report of an intervention should detail not only what was done and by whom, but why and how it was done. This applies equally to the control arms of studies. If claims are made about the effects of an intervention then it is vital to understand not only what is done and why, but exactly what the differences are between experimental and control packages. This paper has described a mechanism for searching for these active ingredients, and has provided the first step in opening the ‘black box’ of what works in terms of midwifery-led antenatal care. Our future work will compare different models of care by exploring in detail those characteristics identified by the QMNC framework as relevant to quality maternal and newborn care. This is essential to enable the active ingredients of a successful intervention to be replicated beyond the research context.

## Abbreviations

PICOS, population, interventions, comparator, outcomes, study design; QMNC, quality maternal and newborn care; RCT, randomised controlled trial; SMART, Scottish midwives advancing research together; SPIO, study design, population, intervention, outcome; SR, systematic review

## References

[1] World Health Organisation (2011). WHO Statement on Antenatal Care.

[2] Dowswell T, Carroli G, Duley L, Gates S, Gulmezoglu AM, Khan-Neelofur D, Piaggio GG (2010). Alternative versus standard packages of antenatal care for low-risk pregnancy. Cochrane Database Syst Rev.

[3] Sandall J, Soltani H, Gates S, Shennan A, Devane D (2013). Midwife-led continuity models versus other models of care for childbearing women. Cochrane Database Syst Rev.

[4] Sandall J, Soltani H, Gates S, Shennan A, Devane D. Midwife-led continuity models versus other models of care for childbearing women. Cochrane Database Syst Rev. 2015(CD004667). DOI: 10.1002/14651858.CD004667 pub5. 10.1002/14651858.CD004667.pub4 26370160

[5] Renfrew MJ, McFadden A, Bastos MH, Campbell J, Channon AA, Cheung NF, Audebert Delage Silva DR, Downe S, Kennedy HP, Malata A (2014). Midwifery and quality care: findings from a new evidence-informed framework for maternal and newborn care. Lancet.

[6] Ten Hoope-Bender P, de Bernis L, Campbell J, Downe S, Fauveau V, Fogstad H, Homer CSE, Kennedy HP, Matthews Z, McFadden A (2014). Improvement of maternal and newborn health through midwifery. Lancet.

[7] Green J, Curtis P, Price H, Renfrew M (2001). Continuing to care.

[8] Carroli G, Villar J, Piaggio G, Khan-Neelofur D, Gulmezoglu M, Mugford M, Lumbiganon P, Farnot U, Bersgjo P, Group WHOACTR (2001). WHO systematic review of randomised controlled trials of routine antenatal care. Lancet.

[9] Requejo J, Victora C, Bryce J, Sci Review Grp C (2014). Data Resource Profile: Countdown to 2015: Maternal, Newborn and Child Survival. Int J Epidemiol.

[10] Allen J, Gamble J, Stapleton H, Kildea S (2012). Does the way maternity care is provided affect maternal and neonatal outcomes for young women? A review of the research literature. Women Birth.

[11] Ickovics J, Kershaw T, Westdahl C, Magriples U, Massey Z, Reynolds H, Schindler Rising S (2007). Group Prenatal Care and Perinatal Outcomes: A Randomized Controlled Trial. Obstet Gynecol.

[12] Davis-Floyd R, Barclay L, Daviss B-A, Tritten J, Davis-Floyd R, Barclay L, Daviss B-A, Tritten J (2009). Introduction. Birth Models That Work.

[13] Dahlen HG, Tracy S, Tracy M, Bisits A, Brown C, Thornton C (2014). Rates of obstetric intervention and associated perinatal mortality and morbidity among low-risk women giving birth in private and public hospitals in NSW (2000-2008): a linked data population-based cohort study. BMJ Open.

[14] Jackson N, Paterson-Brown S (2001). Physical sequelae of caesarean section. Best Pract Res Clin Obstet Gynaecol.

[15] Downe S (2014). Reducing routine interventions during labour and birth: first, do no harm. Cadernos de saude publica.

[16] Blustein J, Liu J (2015). Time to consider the risks of caesarean delivery for long term child health. BMJ-British Med J.

[17] Mangham U, Petrou S, Doyle LW, Draper ES, Marlow N (2009). The cost of preterm birth throughout childhood in England and Wales. Pediatrics.

[18] Petrou S (2005). The economic consequences of preterm birth during the first 10 years of life. BJOG.

[19] Department of Health. Maternity Matters: choice, access and continuity of care in a safe service. In. Edited by Department of Health. London: HMSO; 2007.

[20] National Institute for Health and Care Excellence (2014). Intrapartum Care: Care of Healthy Women and Their Babies During Childbirth (CG190).

[21] Maternity Services Action Group (2011). A Refreshed Framework for Maternity Services.

[22] Care Quality Commission (2013). National findings from the 2013 survey of women’s experiences of maternity care.

[23] McLachlan HL, Forster DA, Davey MA, Farrell T, Gold L, Biro MA, Albers L, Flood M, Oats J, Waldenstrom U (2012). Effects of continuity of care by a primary midwife (caseload midwifery) on caesarean section rates in women of low obstetric risk: the COSMOS randomised controlled trial. Bjog.

[24] Begley C, Devane D, Clarke M, McCann C, Hughes P, Reilly M, Maguire R, Higgins S, Finan A, Gormally S (2011). Comparison of midwife-led and consultant-led care of healthy women at low risk of childbirth complications in the Republic of Ireland: a randomised trial. BMC Pregnancy Childbirth.

[25] Biro MA, Waldenstrom U, Brown S, Pannifex JH (2003). Satisfaction with team midwifery care for low- and high-risk women: a randomized controlled trial. Birth.

[26] Biro MA, Waldenstrom U, Pannifex JH (2000). Team midwifery care in a tertiary level obstetric service: a randomized controlled trial. Birth.

[27] Flint C, Poulengeris P, Grant A (1989). The ‘Know Your Midwife’ scheme--a randomised trial of continuity of care by a team of midwives. Midwifery.

[28] Giles W, Collins J, Ong F, MacDonald R (1992). Antenatal care of low risk obstetric patients by midwives. A randomised controlled trial. MedJAust.

[29] Gu C, Wu X, Ding Y, Zhu X, Zhang Z (2013). The effectiveness of a Chinese midwives’ antenatal clinic service on childbirth outcomes for primipare: a randomised controlled trial. IntJ Nurs Stud.

[30] Harvey S, Rach D, Stainton MC, Jarrell J, Brant R (2002). Evaluation of satisfaction with midwifery care. Midwifery.

[31] Hicks C, Spurgeon P, Barwell F (2003). Changing Childbirth: a pilot project. J Adv Nurs.

[32] Homer CS, Matha DV, Jordan LG, Wills J, Davis GK (2001). Community-based continuity of midwifery care versus standard hospital care: a cost analysis. Aust Health Rev.

[33] Homer CS, Davis GK, Brodie PM, Sheehan A, Barclay LM, Wills J, Chapman MG (2001). Collaboration in maternity care: a randomised controlled trial comparing community-based continuity of care with standard hospital care. Bjog.

[34] North Staffordshire Changing Childbirth Research T (2000). A randomised study of midwifery caseload care and traditional ‘shared-care’. Midwifery.

[35] Rowley MJ, Hensley MJ, Brinsmead MW, Wlodarczyk JH (1995). Continuity of care by a midwife team versus routine care during pregnancy and birth: a randomised trial. Med J Aust.

[36] Tracy SK, Hartz DL, Tracy MB, Allen J, Forti A, Hall B, White J, Lainchbury A, Stapleton H, Beckmann M (2013). Caseload midwifery care versus standard maternity care for women of any risk: M@NGO, a randomised controlled trial. Lancet.

[37] Turnbull D, Holmes A, Shields N, Cheyne H, Twaddle S, Gilmour WH, McGinley M, Reid M, Johnstone I, Geer I (1996). Randomised, controlled trial of efficacy of midwife-managed care. Lancet.

[38] Turnbull D, Shields N, McGinley M (1999). Can midwife-managed units improve continuity of care?. British J Midwifery.

[39] Shields N, Turnbull D, Reid M, Holmes A, McGinley M, Smith LN (1998). Satisfaction with midwife- managed care in different time periods: a randomised controlled trial of 1299 women. Midwifery.

[40] Young D, Shields N, Holmes A (1997). A new style of midwife-managed antenatal care: costs and satisfaction. British J Midwifery.

[41] Waldenstrom U, Brown S, McLachlan H, Forster D, Brennecke S (2000). Does team midwife care increase satisfaction with antenatal, intrapartum, and postpartum care? A randomized controlled trial. Birth.

[42] Waldenstrom U, McLachlan H, Forster D, Brennecke S, Brown S (2001). Team midwife care: maternal and infant outcomes. Aust NZJ Obstet Gynaecol.

[43] Waldenstrom U, Nilsson CA (1994). Experience of childbirth in birth center care. A randomized controlled study. Acta Obstet GynecolScand.

[44] Waldenstrom U, Nilsson CA (1997). A randomized controlled study of birth center care versus standard maternity care: effects on women’s health. Birth.

[45] Waldenstrom U, Nilsson CA, Winbladh B (1997). The Stockholm birth centre trial: maternal and infant outcome. British J Obstetrics Gynaecol.

[46] Walker D, DeMaria L, Gonzalez-Hernandez D, Padron-Salas A, Romero-Alvarez M, Suarez L (2013). Are all skilled birth attendants created equal? A cluster randomised controlled study of nonphysician based obstetric care in primary health care clinics in Mexico. Midwifery.

[47] Wu Z, Viisainen K, Wang Y, Hemminki E (2011). Evaluation of a community-based randomized controlled prenatal care trial in rural China. BMC Health Serv Res.

[48] National Childbirth Trust (2007). NCT Policy Briefing: NICE Intrapartum Care Guideline: Care of Healthy Women and Their Babies During Childbirth.

[49] Hartz DL, Foureur M, Tracy SK (2012). Australian caseload midwifery: the exception or the rule. Women Birth.

[50] Walker DM, DeMaria L, Suarez L, Gonzales D, Romero M, Padron A (2012). Are all skilled birth attendants created equal? evidence from mexico. Int J Gynaecol Obstet.

[51] Flint C: Continuity of care provided by a team of midwives-the Know Your Midwife scheme. In: Robinson S, Thomson AM (eds) 1991:72-103.

[52] Dennis C, Dowswell T. Psychosocial and psychological interventions for preventing postpartum depression. Cochrane Database Syst Rev 2013. (2). doi:10.1002/14651858.CD001134.pub3. 10.1002/14651858.CD001134.pub3 PMC11936315 23450532

[53] Alderdice F, McNeill J, Lynn F (2013). A systematic review of systematic reviews of interventions to improve maternal mental health and well-being. Midwifery.

[54] NHS England. Better Births: Improving outcomes of maternity services in England (the National Maternity Review). In. London: NHS England; 2016.

[55] National Institute for Health and Care Excellence (2007). Intrapartum Care: Care of Healthy Women and Their Babies During Childbirth (CG55).

[56] National Institute for Health and Care Excellence (2015). Safe Midwifery Staffing for Maternity Settings (NG4).

[57] Sandall J, Homer C, Sadler E, Rudisill C, Bourgeault I, Bewley S, Nelson P, Cowie L, Cooper C, Curry C (2011). Staffing In Maternity Units: Getting the Right People in the Right Place at the Right Time. In.

[58] Romano AM, Lothian JA (2008). Promoting, protecting, and supporting normal birth: A look at the evidence. Jognn.

[59] World Health Organisation (2014). The prevention and elimination of disrespect and abuse during facility-based childbirth.

[60] White Ribbon Alliance (2011). Respectful Maternity Care: The Universal Rights of Childbearing Women.

[61] Bowser D, Hill K. Exploring Evidence for Disrespect and Abuse in Facility-based Childbirth: report of a landscape analysis. In: USAID/ TRAction Project. 2010.

[62] Freedman LP, Kruk ME (2014). Disrespect and abuse of women in childbirth: challenging the global quality and accountability agendas. Lancet.

[63] Marshall JL, Spiby H, McCormick F (2015). Evaluating the ‘Focus on Normal Birth and Reducing Caesarean section Rates Rapid Improvement Programme’: A mixed method study in England. Midwifery.

[64] Warren C, Njuki R, Abuya T, Ndwiga C, Maingi G, Serwanga J, Mbehero F, Muteti L, Njeru A, Karanja J, et al. Study protocol for promoting respectful maternity care initiative to assess, measure and design interventions to reduce disrespect and abuse during childbirth in Kenya. BMC Pregnancy Childbirth 2013;13. doi: 10.1186/1471-2393-13-21. 10.1186/1471-2393-13-21 PMC3559298 23347548

[65] Sacks E, Kinney MV (2015). Respectful maternal and newborn care: building a common agenda. Reprod Health.

[66] Hastings-Tolsma M, Nolte AGW (2014). Reconceptualising failure to rescue in midwifery: A concept analysis. Midwifery.

[67] Fullerton JT, Thompson JB, Johnson P (2013). Competency-based education: The essential basis of pre-service education for the professional midwifery workforce. Midwifery.

[68] ICM International Definition of the Midwife. [http://www.internationalmidwives.org/assets/uploads/documents/Definition%20of%20the%20Midwife%20-%202011.pdf]. Accessed 10 Jul 2016.

[69] National Collaborating Centre for Primary Care (2006). Postnatal Care: Routine Postnatal care of Women and their Babies.

[70] National Federation of Women’s Institutes / National Childbirth Trust (2013). Support Overdue: Women’s Experiences of Maternity Services.

[71] Henderson J, Redshaw M (2011). Midwifery factors associated with successful breastfeeding. Child: Care, Health Dev.

[72] McDonald SJ, Henderson JJ, Faulkner S, Evans SF, Hagan R (2010). Effect of an extended midwifery postnatal support programme on the duration of breast feeding: a randomised controlled trial. Midwifery.

[73] Morton J (2014). How midwives can help with perinatal depression. pract midwife.

[74] Hundley VA, Cruickshank FM, Milne JM, Glazener CM, Lang GD, Turner M, Blyth D, Mollison J (1995). Satisfaction and continuity of care: staff views of care in a midwife-managed delivery unit. Midwifery.

